# Association of macronutrients intake distribution with osteoarthritis risk among adults in NHANES, 2013–2016

**DOI:** 10.3389/fnut.2023.976619

**Published:** 2023-03-23

**Authors:** Peng Peng, Shihua Gao, Fangjun Xiao, Mincong He, Weiuhua Fang, Yunqi Zhang, Qiushi Wei

**Affiliations:** ^1^Guangzhou University of Chinese Medicine, Guangzhou, China; ^2^Guangdong Research Institute for Orthopedics and Traumatology of Chinese Medicine, Guangzhou, China; ^3^Department of Orthopaedics, The Third Affiliated Hospital, Guangzhou University of Chinese Medicine, Guangzhou, China; ^4^Department of Pharmacy, Guangzhou Institute of Dermatology, Guangzhou, China

**Keywords:** osteoarthritis, macronutrients, NHANES, dietary pattern, cross-sectional study

## Abstract

The association between dietary macronutrient distribution and the risk of OA remains unknown. We aimed to evaluate how dietary macronutrient distribution was correlated with the risk of OA in US adults. We performed a cross-sectional study consisting of 7,725 participants from National Health and Nutrition Examination Survey (NHANES) 2013–2016. Dietary macronutrient intake and OA status were assessed by using dietary recall method and self-reported questionnaire, respectively. We evaluated the association between dietary macronutrient distribution and the risk of OA using multivariate regression models. We conducted the isocaloric substitution analysis using the multivariate nutrient density method. Higher percentage of energy intake from fat was associated with higher risk of OA [OR = 1.05 (95% CI, 1.00, 1.09); *P* = 0.034]. No significant correlation was observed between the percentage of energy intake from carbohydrate or protein and risk of OA. Isocaloric substitution analysis revealed that only the substitution between fat and carbohydrate was significantly associated with the risk of OA [OR = 1.05 (95% CI, 1.003 to 1.09); *P* = 0.037]. Our findings suggested that a diet with low percentage of energy intake from fat may be beneficial in the prevention of OA. Further prospective cohort studies are needed to assess our results.

## Introduction

Osteoarthritis (OA) is a high incidence joint disease characterized by degeneration in joint tissue structure, which often causes chronic pain and joint dysfunction among the patients (1). Globally, more than 360 million people are currently suffering from this disease, and the prevalence of OA keeps increasing yearly (2). Approximately 27 million US adults have clinical OA in 2005, an increase from 21 million in 1995 (3). OA has a huge impact economically, in addition to its effect on health. In the United States, the annual cost of joint replacement for OA was estimated at $22.6 billion, and the job-related OA cost was approximately $13.2 billion (4, 5).

The pathogenesis of OA is multifactorial, involving inflammatory, mechanical, and metabolic factors, which can ultimately lead to synovial inflammation and structural destruction of the joint (6). Accumulating evidence has shown that nutrition intake is involved in the development or progression of OA (7–9). Dietary patterns has also been studied in the occurrence and prevention of OA. A large prospective study with 2,842 participants found that higher adherence to western dietary pattern was associated with higher risk of knee OA (10). A cross-sectional study revealed that healthy dietary patterns were related to reduced joint symptoms but dietary patterns were not related to joint structural change in OA patients (11). In a 4-years longitudinal follow-up cohort study, researchers demonstrated that participants with a higher adherence to Mediterranean diet had a lower risk of pain worsening and symptomatic knee OA (12).

Recently, the importance of the proportions of macronutrients intake is also emphasized in the development of chronic diseases (13–15). However, there are limited data on the association between the proportions of macronutrient intake with the risk of OA. High intake of total fat and saturated fatty acids (SFA) may be related to increased progression of structural knee OA, whereas higher intake of mono- and poly-unsaturated fatty may be related to reduced radiographic progression (16). However, only fat intake in macronutrient was analyzed, and other nutrients (carbohydrate and protein) were not adjusted into the model, which would make the interpretation of the results become difficult because the estimated effect of fat may depend on other nutrients (carbohydrate and protein) it replaces. More studies are needed to better investigate the relationship between dietary macronutrient distribution and risk of OA.

To fill the aforementioned knowledge gaps, we aimed to explore the association between dietary macronutrient distribution and risk of OA in US adults using data from the National Health and Nutrition Examination Survey (NHANES) database.

## Materials and methods

### Study population

**FIGURE 1:**
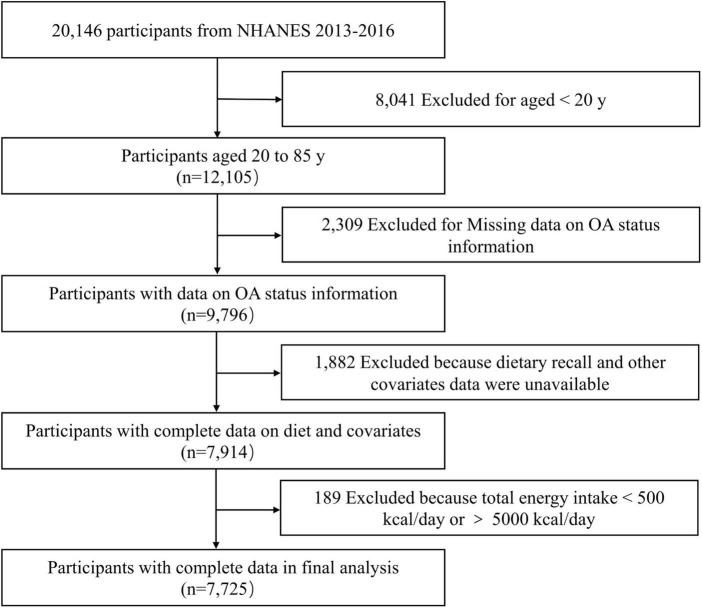
Flowchart of the participants selection.

The NHANES database is an ongoing population-based national survey focusing on the nutrition and health of the American population. The NHANES database is available publicly at www.cdc.gov/nchs/nhanes. Data from 2013 to 2016 in NHANES were combined in this study. We investigated the links between macronutrient distribution with risk of OA in adult participants, including 12,105 participants aged over 20 years. Participants with missing value for arthritis status information (*n* = 2,309), dietary recall and other covariates (*n* = 1,882), were excluded. After the exclusion of 189 participants with unusually low or high total energy intake (< 500 kcal/day or > 5,000 kcal/day), 7,725 participants were enrolled, including 1,039 OA patients (Figure 1).

### Assessment of OA status

Osteoarthritis (OA) status was determined by a questionnaire survey (17). Participants were asked: “Has a doctor or other health professional ever told you that you have arthritis?” Those who answered “no” were defined as without OA. If the answer is “yes,” the patients will be further to answer a follow-up question, “Which type of arthritis was it?” Those who self-reported “osteoarthritis” were defined as with OA.

### Assessment of macronutrients intake distribution

Nutrient intake information was collected through two non-consecutive 24-h dietary recalls (18). The “automated multiple pass method” was used to improve the precision of food recall, the steps of this methods are as follows: finishing a self-reported food list, probing for forgotten foods, collecting details of foods, and final probing for any other foods. To avoid the difference in dietary intake between weekdays and weekends, only recalls of weekdays were chosen. If both recalls were recorded on weekdays, the first recall would be chosen. A standardized measuring guide was also used to quantify the amount of food items. Total consumption of protein, carbohydrate, and fat were calculated according to the recorded food items. Daily total energy intake was generated by summing the calories from protein, carbohydrate, and fat (1 g protein = 4 kcal, 1 g carbohydrate = 4 kcal, 1 g fat = 9 kcal) (19). Macronutrients distribution was further calculated as follows:


(1) $$\mathrm{Carbohdrate}\mathrm{intake}\left(\%\right)=\frac{4\,\mathrm{kcal}/\text{g}\;\times \;\mathrm{daily}\,\mathrm{carbohdrate}\,\mathrm{intake}}{\mathrm{daily}\,\mathrm{total}\,\mathrm{energy}\,\mathrm{intake}\,\left(\text{kcal}\right)}\times 100\%$$

(2) $$\mathrm{Protein}\mathrm{intake}\left(\%\right)=\frac{4\,\mathrm{kcal}/\text{g}\;\times \;\mathrm{daily}\,\mathrm{protein}\,\mathrm{intake}}{\mathrm{daily}\,\mathrm{total}\,\mathrm{energy}\,\mathrm{intake}\,\left(\text{kcal}\right)}\times 100\%$$

(3) $$\mathrm{Fat}\mathrm{intake}\left(\%\right)=\frac{9\,\mathrm{kcal}/\text{g}\;\times \;\mathrm{daily}\,\mathrm{fat}\,\mathrm{intake}}{\mathrm{daily}\,\mathrm{total}\,\mathrm{energy}\,\mathrm{intake}\,\left(\text{kcal}\right)}\times 100\%$$

### Covariates

To assess physical activity, weekly metabolic equivalent (MET) minute aggregated scores were calculated for each participant (20). Referring to the recommended method of NHANES, weekly MET-minutes were calculated as follows: [4.0 MET scores × (weekly minutes of moderate work-related activity + weekly minutes walking or bicycling for transportation) + weekly minutes of moderate leisure-time physical activity] + [8.0 MET scores × (weekly minutes of vigorous work-related activity + weekly minutes of vigorous leisure-time physical activity)]. Using the calculated MET-minutes, participants were categorized into inactive (< 600 MET-minute/week), moderately active (600–3,000 MET-minute/week), and highly active (> 3,000 MET-minute/week). Dietary fiber intake were collected based on dietary recall and supplement use recall. Diabetes status was determined based on a questionnaire, in which the patient answers the question “Have you ever been told by a doctor or health professional that you have diabetes or sugar diabetes?” Those who answered “yes” were defined as with self-report diabetes status. Those who answered “no” or “borderline” were defined as without self-report diabetes status. The other covariates included age, race/ethnicity, education, body mass index, total protein, total cholesterol, serum calcium, and serum 25-hydroxyvitamin D. The examination parts related to clinical and laboratory evaluations were all carried out by well-trained medical experts. Information on each variable and acquisition process are publicly available at www.cdc.gov/nchs/nhanes.

### Statistical analysis

Continuous variables were presented as medians and inter quartile ranges and categorical variables as percentages. We used the Kruskal-Wallis test for continuous variables and the χ2 test for categorical variables to assess the characteristics of the participants by self-reported OA status. Multivariate logistic regression analyses were performed to evaluate the relationship between macronutrient distribution with OA risk with odds ratio (OR) and corresponding 95% confidence interval (CI). Three models were constructed, as follows: crude model, no adjustment for covariates; model 1, adjusted for age, gender, and race/ethnicity; model 2, additionally adjusted for education, self-reported diabetes, body mass index, total protein, total cholesterol, serum calcium, serum 25-hydroxyvitamin D, metabolic equivalent task minutes, dietary fiber intake and total energy intake. Sensitivity analysis was conducted by stratifying participants according to different intake levels of macronutrients with reference to the acceptable macronutrient distribution ranges (21). The trend test was calculated by treating the intake of each category of macronutrients as a continuous variable in multivariable models.

We performed isocaloric substitution analysis to assess whether substituting certain type of macronutrient (as 5% of energy) with another is associated with OA risk using multivariate nutrient density method (e.g., replacing 5% of the energy intake of carbohydrate with fat intake while leaving protein intake unchanged) (22). Subgroup analyses were also conducted stratified by different age, gender, BMI, self-reported diabetes, total cholesterol, and physical activity level. A two-sided *P*-value < 0.05 was considered statistically significant. Statistical analyses were done with the EmpowerStats (^1^ X&Y Solutions, Inc., Boston, MA, USA) and statistical software packages R (^2^ The R Foundation).

## Results

### Characteristics of participants

The characteristics of participants were presented in Table 1. Among the 7,725 participants, 1,039 were diagnosed with OA. Compared with the non-OA group, the OA group was older, and had a higher proportion of women than men (64.58 versus 49.28%). Participants with OA or non-OA were similar in education level and serum calcium, while race/ethnicity, self-reported diabetic status, BMI, total protein, serum total cholesterol, serum 25-hydroxyvitamin D, and MET minutes were significantly different between these two groups. For dietary intake, total energy, protein, carbohydrate, and dietary fiber intake were all similar between OA and non-OA groups. The percentage of energy intake from fat was significantly higher in the OA group.

**TABLE 1:** Characteristics of participants stratified by self-reported osteoarthritis (OA) status (*N* = 7,725).

Characteristics	Total	OA	Non-OA	*P*-value
N	7,725	1,039	6,686	
Age (years)	45 (32–61)	65 (55–73)	42 (31–57)	<0.001
Gender, n (%)				<0.001
Male	3759 (48.66)	368 (35.42)	3391 (50.72)	
Female	3966 (51.34)	671 (64.58)	3295 (49.28)	
Race/Ethnicity, n (%)				<0.001
Non-hispanic White	3019 (39.08)	637 (61.31)	2382 (35.63)	
Non-hispanic Black	1458 (18.87)	147 (14.15)	1311 (19.61)	
Non-hispanic Asian	907 (11.74)	46 (4.43)	861 (12.88)	
Mexican American	1224 (15.84)	94 (9.05)	1130 (16.90)	
Other hispanic	860 (11.13)	77 (7.41)	783 (11.71)	
Other	257 (3.33)	38 (3.66)	219 (3.28)	
Education, n (%)				0.336
Lower than high school	1541 (26.97)	186 (17.9)	1355 (20.27)	
High school or equivalent	1692 (21.90)	216 (20.79)	1476 (22.08)	
More than high school	4492 (51.13)	637 (61.31)	3855 (57.65)	
Self-reported diabetes, n (%)				<0.001
Yes	933 (12.08)	249 (23.97)	684 (10.23)	
No	6792 (87.92)	790 (76.03)	6002 (89.77)	
BMI (kg/m^2^)	27.8 (24.2–32.4)	30.4 (25.9–35.6)	27.5 (23.9–31.9)	<0.001
Total protein (g/dL)	71 (68–74)	70 (67–73)	72 (69–75)	<0.001
Total cholesterol (mg/dL)	186 (161–214)	190 (162–220)	186 (161–213)	0.019
Serum calcium (mg/dL)	9.4 (9.2–9.6)	9.37 (9.2–9.6)	9.4 (9.22–9.6)	0.077
Serum 25-hydroxyvitamin D (nmol/L)	61.6 (44.8–79.8)	75.3 (56.3–95.75)	59.85 (43.6–77.3)	<0.001
MET minutes per week	400 (60–1,200)	180 (60–720)	480 (80–1,200)	<0.001
Total energy intake (kcal/d)	1953 (1,463–2,568)	1767 (1,309–2,357.5)	1978 (1,484–2,602)	<0.001
Protein intake (%)	15.2 (12.4–18.7)	15.15 (12.35–18.25)	15.25 (12.45–18.85)	0.212
Carbohydrate intake (%)	48.25 (41–55.5)	48.25 (41.2–55.25)	48.3 (40.9–55.5)	0.987
Fat intake (%)	34.3 (28.2–40.1)	34.9 (29.2–40.55)	34.15 (28.05–40)	0.004
Dietary fiber intake (g/d)	15 (9.8–21.9)	14.70 (9.5–21.35)	15 (9.8–22)	0.066

Median (inter quartile range) for continuous variables and *P*-value was calculated by Kruskal-Wallis test. N (%) for categorical variables and *P*-value was calculated by weighted chi-square test. BMI, body mass index; MET, metabolic equivalent task.

### Macronutrients distribution and OA risk

Table 2 showed the association between macronutrients intake distribution and risk of OA. A negative association between fat intake with risk of OA was found in the crude model [OR = 0.95 (95% CI, 0.92, 0.99); *P* = 0.010]. However, higher fat intake was associated with higher risk of OA [OR = 1.05 (95% CI, 1.00, 1.09); *P* = 0.034] after full adjustment. Fat intake level above reference range (> 35% energy) was highly correlated with higher risk of OA [OR = 1.36 (0.96, 1.92); *P* = 0.042]. Linear trend was shown across the intake levels of fat. Intake of carbohydrate was not significantly associated with OA risk [OR = 0.98 (0.95, 1.02); *P* = 0.261]. Meanwhile, no significant association was observed between protein intake and the risk of OA [OR = 1.03 (0.95, 1.011); *P* = 0.489].

**TABLE 2:** Association between macronutrients distribution and the risk of osteoarthritis (OA) among 7,725 participants from 2013 to 2016 National Health and Nutrition Examination Survey (NHANES).

Nutrients distribution	OA OR (95% CI)		
	Crude	Model 1	Model 2
Carbohydrate intake (5% increase)	0.99 (0.97, 1.02)	1.003 (0.97, 1.04)	0.98 (0.95, 1.02)
**Carbohydrate intake levels**			
Below reference (<45%)	[Ref]	[Ref]	[Ref]
Reference intake (45–65%)	0.98 (0.85, 1.12)	1.05 (0.90, 1.22)	0.98 (0.83, 1.15)
Above reference (>65%)	0.92 (0.70, 1.21)	0.88 (0.65, 1.20)	0.81 (0.59, 1.12)
P for trend	0.579	0.934	0.366
Protein intake (5% increase)	1.05 (0.99, 1.11)	1.02 (0.96, 1.10)	1.03 (0.95, 1.11)
**Protein intake levels**			
Below reference (<10%)	[Ref]	[Ref]	[Ref]
Reference intake (10–35%)	1.17 (0.95, 1.45)	1.33 (1.04, 1.68)	1.40 (1.08, 1.80)
Above reference (>35%)	1.20 (0.60, 2.41)	1.09 (0.50, 2.39)	1.11 (0.49, 2.48)
P for trend	0.150	0.042	0.024
Fat intake (5% increase)	0.95 (0.92, 0.99)	1.001 (0.96, 1.04)	1.05 (1.00, 1.09)
**Fat intake levels**			
Below reference (<20%)	[Ref]	[Ref]	[Ref]
Reference intake (20–35%)	0.90 (0.67, 1.21)	1.07 (0.77, 1.48)	1.15 (0.82, 1.61)
Above reference (>35%)	0.79 (0.59, 1.06)	1.09 (0.78, 1.51)	1.36 (0.96, 1.92)
P for trend	0.026	0.649	0.015

Crude model: No covariate was adjusted. Model 1: Age, gender, race/ethnicity were adjusted. Model 2: Additionally adjusted for education, self-reported diabetes, body mass index, total protein, total cholesterol, serum calcium, serum 25-hydroxyvitamin D, metabolic equivalent task minutes, dietary fiber intake, and total energy intake. CI, confidence interval; OR, odds ratio.

### Isocaloric substitution analysis

Table 3 presented the analysis of isocaloric substitution of macronutrients. Isocaloric substitution of carbohydrate by fat was associated with higher risk of OA [OR = 1.05 (95% CI, 1.003 to 1.09); *P* = 0.037], whereas replacement of protein with fat was not significantly associated with risk of OA [OR = 1.06 (95% CI, 1.00 to 1.12); *P* = 0.062]. No significant association between carbohydrate and protein substitution with OA risk was detected.

**TABLE 3:** Association between isocaloric substitution of macronutrients and the risk of osteoarthritis (OA) among 7,725 participants from 2013 to 2016 National Health and Nutrition Examination Survey (NHANES).

Isocaloric substitution (5% energy)	OA OR (95% CI)		
	Crude	Model 1	Model 2
Protein substituting carbohydrate	1.05 (0.99, 1.11)	1.02 (0.96, 1.10)	1.02 (0.95, 1.10)
Fat substituting carbohydrate	0.95 (0.92, 0.99)	1.001 (0.96, 1.04)	1.05 (1.003, 1.09)
Fat substituting protein	0.95 (0.92, 0.99)	1.003 (0.95, 1.06)	1.06 (0.99, 1.12)

Crude model: No covariate was adjusted. Model 1: Age, gender, race/ethnicity were adjusted. Macronutrient intakes also entered multivariate regression models apart from the substituted one. Model 2: Additionally adjusted for education, self-reported diabetes, body mass index, total protein, total cholesterol, serum calcium, serum 25-hydroxyvitamin D, metabolic equivalent task minutes, dietary fiber intake, and total energy intake. CI, confidence interval; OR, odds ratio.

**FIGURE 2:**
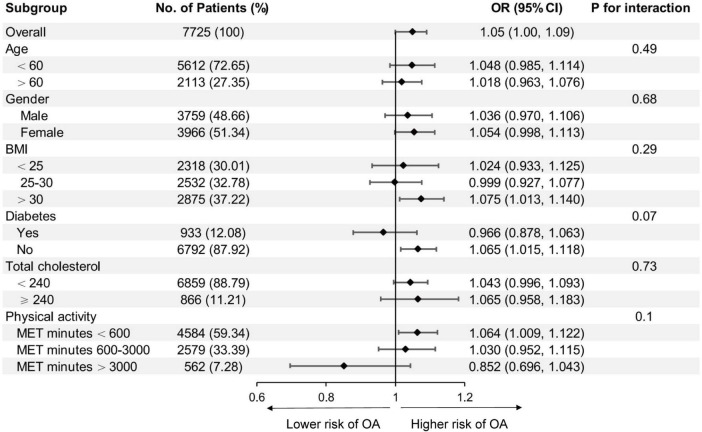
Association between isocaloric substitution of fat for carbohydrate intake with osteoarthritis (OA) risk in different subgroups. BMI, body mass index; MET, metabolic equivalent task. Age, gender, race/ethnicity, education, diabetes, body mass index, total protein, total cholesterol, serum calcium, serum 25-hydroxyvitamin D, metabolic equivalent task minutes, dietary fiber intake, total energy intake, and protein intake were adjusted (the stratified variable was omitted from the model).

Subgroup analysis was conducted to examine whether the association between isocaloric fat-carbohydrate substitution and the risk of OA were consistent among different population groups (Figure 2). When stratified by age, gender, BMI, diabetes, total cholesterol, or physical activity level, no statistically significant difference was observed.

## Discussion

In the current study, we found that higher percentage of energy intake from fat was associated with higher risk of OA. No significant correlation was observed between the percentage of energy intake from carbohydrate or protein and the risk of OA. Isocaloric substitution analysis indicated that only the substitution between fat and carbohydrate was significantly correlated with the incidence of OA. The replacement of carbohydrate with fat for every 5% of energy intake was correlated with 5% higher risk of OA. The association between fat-carbohydrate isocaloric substitution and the risk of OA remained consistent in subgroup analysis, indicating the correlation was not modified by age, gender, BMI, diabetes, total cholesterol, and physical activity levels.

Osteoarthritis (OA) is recognized as a multifactorial inflammatory disease, including obesity, synovitis, and systemic inflammatory mediators (10). Numerous studies have suggested that diet nutrients could related to inflammation markers (23–25), which may lead to OA progression. Western dietary pattern has been demonstrated to be associated with chronic inflammatory process that was involved in many chronic degenerative diseases (26). A systematic review reported that adherence to a western dietary pattern was associated with higher levels of pro-inflammatory biomarkers such as interleukin (IL)-6, c-reactive protein (CRP), and tumor necrosis factor-alpha (TNF-α) (27). Western diet can induced gut-derived inflammation, which disrupts mechanisms for maintaining energy homeostasis and lead to obesity and subsequent metabolic disease (28). In addition, two studies investigated the data from the Osteoarthritis Initiative (OAI) suggested that adopting western dietary pattern was associated with increased risk and radiographic progression of knee OA (10, 29). In general, western dietary pattern is correlated with higher risk of OA.

Western dietary pattern is characterized by high-fat dairy products, refined grains, and large consumption of red meat. Some findings revealed that lipids can interact with chondrocytes and articular cartilage, leading to inflammation and cartilage degradation (30). With diet influencing systemic lipid levels (31), dietary fat may play a role in the development and progression of OA. A Multicenter Osteoarthritis Study (MOST) detected a positive association between the n-6 polyunsaturated fatty acid (PUFA) with synovitis in OA but an inverse relationship between total plasma n-3 PUFA (32). Western dietary pattern contain a higher levels of n-6 PUFAs than n-3 PUFAs, which predisposes to inflammation (33). A prospective cohort study reported that higher intake of total fat and saturated fatty acids (SFA) may be related to increased progression of structural knee OA (16), which is consistent with our findings.

Based on our results and previous studies, higher fat consumption may contribute to the development of OA. Several animal studies have shown a high link between dietary fat intake and OA. In rabbit model, increased dietary fat was associated with changes in cartilage and appears to be a risk factor for the development of OA (34). A high fat diet seems to induce or exacerbate the progression of OA in mice by causing metabolic changes and systemic inflammation (35). In another mice study, a very high fat diet increased knee OA scores and the levels of serum leptin, adiponectin, IL-8, and IL-1α (36).

Limited study available regarding the association between carbohydrate and protein intake with the prevalence of OA. In our study, no significant correlation was observed between the percentage of energy intake from carbohydrate or protein with the risk of OA. Interestingly, we found that isocaloric replacement of carbohydrate with fat was associated with the incidence of OA, which may indicated that diet with high percentage of carbohydrate intake coupled with low percentage of fat intake would be beneficial in the prevention of OA. More clinical and basic experiments are needed to prove it.

The strength of this study is that the NHANES database contains representative samples of the multi-ethnic population. In addition, the large sample size allows us to better conduct subgroup analyses. In terms of limitations, first, the nature of the cross-sectional design makes it difficult to determine the causal link between macronutrients intake and risk of OA. Second, the intake of each macronutrient was obtained according to one weekday 24 h dietary recall, which may bias the estimation of usual dietary intake. Third, we used self-reported disease status, making our data susceptible to recall and information biases. In addition, the association of specific types of nutrients with OA has not been studied in the current study. For example, we did not assess the percentage intake of saturated fat or unsaturated fat, because individual types of fat were calculated different from total consumption and the sum of all types of fat was not equal to total consumption. Finally, though we adjusted for several potential confounding variables associated with dietary intake and OA, residual confounding is possible.

## Conclusion

In summary, we found that higher percentage of energy intake from fat was associated with higher risk of OA, while the consumption of carbohydrates and protein were not significantly associated with OA. Isocaloric substitution analysis further indicated that only the substitution between fat and carbohydrate was significantly associated with OA risk. Our findings suggested that a diet with low percentage of energy intake from fat may be beneficial in the prevention of OA. Further prospective cohort studies are needed to assess our results.

## Data availability statement

Publicly available datasets were analyzed in this study. This data can be found here: www.cdc.gov/nchs/nhanes.

## Ethics statement

The studies involving human participants were reviewed and approved by board of the National Center for Health Statistics. The patients/participants provided their written informed consent to participate in this study. Written informed consent was obtained from the individual(s) for the publication of any potentially identifiable images or data included in this article.

## Author contributions

PP and SG conceived the idea of this study. PP and FX wrote the manuscript. WF and MH collected the data and performed the statistical analysis. YZ and QW reviewed the data and revised the manuscript. All authors contributed toward data analysis, drafting and critically revising the manuscript, agreed to be accountable for all aspects of the work, read, and approved the final manuscript.

## Footnotes

1
http://www.empowerstats.com

2
http://www.R-project.org
